# Effect of a mass radio campaign on family behaviours and child survival in Burkina Faso: a repeated cross-sectional, cluster-randomised trial

**DOI:** 10.1016/S2214-109X(18)30004-4

**Published:** 2018-02-09

**Authors:** Sophie Sarrassat, Nicolas Meda, Hermann Badolo, Moctar Ouedraogo, Henri Some, Robert Bambara, Joanna Murray, Pieter Remes, Matthiew Lavoie, Simon Cousens, Roy Head

**Affiliations:** a Centre for Maternal, Adolescent, Reproductive and Child Health (MARCH), London School of Hygiene & Tropical Medicine, London, UK; b Centre Muraz, Bobo Dioulasso, Burkina Faso; c Africsanté, Bobo Dioulasso, Burkina Faso; d Direction Générale des Études et des Statistiques Sectorielles (DGESS), Ministère de la Santé, Ouagadougou, Burkina Faso; e Development Media International, London, UK; f Development Media International, Ouagadougou, Burkina Faso

## Abstract

**Background:**

Media campaigns can potentially reach a large audience at relatively low cost but, to our knowledge, no randomised controlled trials have assessed their effect on a health outcome in a low-income country. We aimed to assess the effect of a radio campaign addressing family behaviours on all-cause post-neonatal under-5 child mortality in rural Burkina Faso.

**Methods:**

In this repeated cross-sectional, cluster randomised trial, clusters (distinct geographical areas in rural Burkina Faso with at least 40 000 inhabitants) were selected by Development Media International based on their high radio listenership (>60% of women listening to the radio in the past week) and minimum distances between radio stations to exclude population-level contamination. Clusters were randomly allocated to receive the intervention (a comprehensive radio campaign) or control group (no radio media campaign). Household surveys were performed at baseline (from December, 2011, to February, 2012), midline (in November, 2013, and after 20 months of campaigning), and endline (from November, 2014, to March, 2015, after 32 months of campaigning). Primary analyses were done on an intention-to-treat basis, based on cluster-level summaries and adjusted for imbalances between groups at baseline. The primary outcome was all-cause post-neonatal under-5 child mortality. The trial was designed to detect a 20% reduction in the primary outcome with a power of 80%. Routine data from health facilities were also analysed for evidence of changes in use and these data had high statistical power. The indicators measured were new antenatal care attendances, facility deliveries, and under-5 consultations. This trial is registered with ClinicalTrial.gov, number NCT01517230.

**Findings:**

The intervention ran from March, 2012, to January, 2015. 14 clusters were selected and randomly assigned to the intervention group (n=7) or the control group (n=7). The average number of villages included per cluster was 34 in the control group and 29 in the intervention group. 2269 (82%) of 2784 women in the intervention group reported recognising the campaign's radio spots at endline. Post-neonatal under-5 child mortality decreased from 93·3 to 58·5 per 1000 livebirths in the control group and from 125·1 to 85·1 per 1000 livebirths in the intervention group. There was no evidence of an intervention effect (risk ratio 1·00, 95% CI 0·82–1·22; p>0·999). In the first year of the intervention, under-5 consultations increased from 68 681 to 83 022 in the control group and from 79 852 to 111 758 in the intervention group. The intervention effect using interrupted time-series analysis was 35% (95% CI 20–51; p<0·0001). New antenatal care attendances decreased from 13 129 to 12 997 in the control group and increased from 19 658 to 20 202 in the intervention group in the first year (intervention effect 6%, 95% CI 2–10; p=0·004). Deliveries in health facilities decreased from 10 598 to 10 533 in the control group and increased from 12 155 to 12 902 in the intervention group in the first year (intervention effect 7%, 95% CI 2–11; p=0·004).

**Interpretation:**

A comprehensive radio campaign had no detectable effect on child mortality. Substantial decreases in child mortality were observed in both groups over the intervention period, reducing our ability to detect an effect. This, nevertheless, represents the first randomised controlled trial to show that mass media alone can change health-seeking behaviours.

**Funding:**

Wellcome Trust and Planet Wheeler Foundation.

## Introduction

Scenario-based projections suggest that, to achieve the Sustainable Development Goal (SDG) target of 25 or fewer under-5 deaths per 1000 livebirths by 2030, about two-thirds of all sub-Saharan African countries will need to accelerate progress in reducing under-5 deaths.^1^ Poor coverage of effective interventions for preventing child deaths has been attributed to weaknesses in both provision of and demand for services.^2^ While much effort towards achieving the Millennium Development Goals has focused on health systems and the supply side,^3^ including community case management of childhood illnesses,^4^, ^5^ less attention has been paid to increasing demand for services. However, it has been acknowledged that behaviour change has an important part to play in enhancing child survival in low-income and middle-income countries.^6^

**Research in context**

**Evidence before this study**

Four reviews, done before this study, concluded that targeted, well executed mass media campaigns can have small to moderate effects not only on health knowledge, beliefs and attitudes, but on behaviours as well (Grilli et al, 2001; Hornick, 2002; Noar, 2006; Bala et al, 2008). However, much of the evidence for an effect comes from non-randomised designs and the limited number of randomised studies that have been reported have often failed to demonstrate an effect. Hornick (2002) has suggested that in many of the randomised trials the exposure to the media was too small to result in an effect. Development Media International's (DMI) experiences in delivering mass media campaign corroborate this crucial implementation principle and indicate that implementation at sufficient scale and intensity is the most important (Head et al, 2015). However, evaluations of the DMI's Saturation+ approach, prior to this study, relied on pre-post designs and on self-reported knowledge or behaviours only. Using the Lives Saved Tool, DMI predicted that a sustained, comprehensive campaign of sufficient scale and intensity could reduce under-5 mortality by between 16% and 23% during the third and subsequent years of campaigning through increases in coverage of key life-saving interventions (Head et al, 2015).

**Added value of this study**

To our knowledge, this study was the first attempt to conduct a randomised controlled trial to test the effect of mass media on a health outcome in a low-income country. From March, 2012, to January, 2015, DMI implemented a comprehensive, high intensity radio campaign to address key family behaviours for improving under-5 child survival in Burkina Faso. Using a repeated cross-sectional, cluster-randomised design, we report on the effect of the campaign on child mortality and family behaviours after 32 months of campaigning.

**Implications of all the available evidence**

The available evidence supports the view that mass media campaigns can lead to changes in some behaviours linked to child survival. However, some behaviours are likely to be less amenable to change than others. Furthermore, that some mass media campaigns can produce changes in behaviour should not be interpreted as meaning that any and every mass media campaign can change behaviour. The “dose” delivered and received by the target audience as well as the quality of the messages are likely to be key determinants of the effectiveness of any campaign. These findings have important policy implications, suggesting that saturation-based media campaigns should be prioritised by governments and belong in the mainstream of public health interventions.

Behaviour change interventions encompass a wide range of approaches including interpersonal-based, community-based, media, and social marketing approaches. Compared with other approaches, mass media campaigns have the potential to reach a large audience at relatively low cost. A recent review of evaluations of mass media interventions for child-survival-related behaviours done between 1960 and 2013 in low-income and middle-income countries concluded that so-called media-centric campaigns can positively affect a wide range of child health behaviours.^7^ Of the 32 evaluations that relied on moderate to stronger designs, all but six were reported to show some positive effects on behaviours. However, the researchers acknowledged likely publication bias towards successful campaigns. Additionally, all but six evaluated programmes included interpersonal communication components or implementation of community-based activities, but none could disentangle the effect of different components. To our knowledge, there have been no attempts to do a randomised controlled trial to test the effect of mass media on any health outcome in a low-income country.

From March, 2012, to January, 2015, Development Media International (DMI) implemented a comprehensive radio campaign to address key family behaviours for improving under-5 child survival in Burkina Faso.^8^ In 2010, Burkina Faso ranked 161 of 169 countries in United Nations Development Programme's Human Development Index with 44% of the population living below the poverty line and 77% living in rural areas.^9^ The under-5 mortality rate was high, estimated at approximately 114 deaths per 1000 livebirths in 2010, with malaria, pneumonia, and diarrhoea the leading causes of child death.^10^ Burkina Faso was chosen both for its high child mortality before the campaign and its unique media landscape (community FM radio stations with limited transmission range and relatively high listenership in rural areas but very limited national radio listenership). We have previously reported on the coverage of the campaign and its effect on behaviours at midline (in November, 2013, and after 20 months of campaigning).^11^ Here we report on the effect of the campaign on child mortality and behaviours at endline—ie, after 32 months of campaigning.

## Methods

### Study design

This study was done using a repeated cross-sectional cluster randomised design by an independent team from the London School of Hygiene & Tropical Medicine and Centre Muraz in Burkina Faso. The intervention and evaluation design have been described previously.^8^, ^9^, ^10^, ^11^, ^12^ Briefly, women of reproductive age and caregivers of children younger than 5 years were the main targets of the campaign, which covered 17 behaviours along the continuum of care. Women were told the surveys were about their children's health, without any mention of the radio campaign, and they recorded their consent to participate in the survey on a Personal Digital Assistant. The study was approved by the ethics committees of the Ministry of Health of Burkina Faso and the London School of Hygiene & Tropical Medicine.

### Randomisation and masking

Of 19 distinct geographical areas, 14, each centred around a community FM radio station, were selected by DMI based on their high listenership (above 60% of women listening to the radio in the past week) and minimum distances between radio stations to exclude population-level contamination. For evaluation purposes, clusters around each radio station were identified using the last national census to provide an evaluation population of about 40 000 inhabitants per cluster. We included villages located around the selected community radio station, with a good radio signal but limited access to television (and thus more likely to listen to the radio). We did this by excluding communities likely to be served by the electricity grid—ie, the towns from which the selected radio stations were broadcast, villages within 5 km of these towns, other villages with electricity or with a population larger than 5000 inhabitants (and likely to be a priority for the national electrification programme).

**Figure 1:**
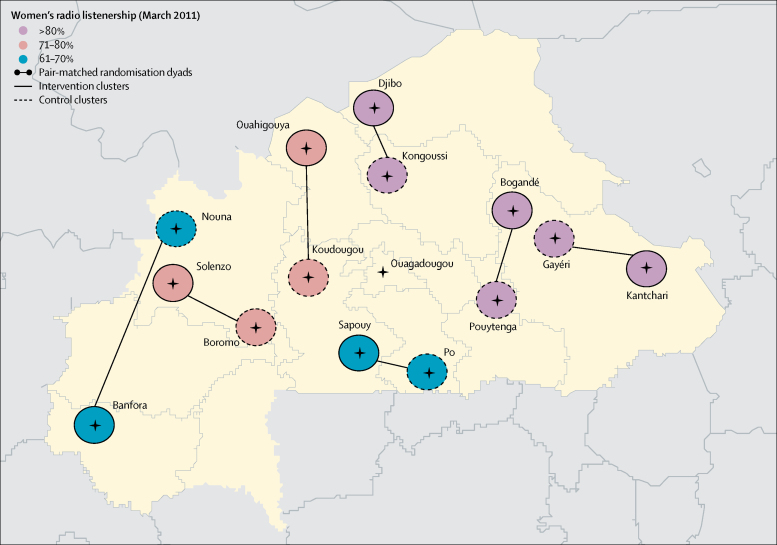
Pair-matched randomisation based on geography and radio penetration rate

Seven clusters were then randomly allocated to receive the intervention or control using pair-matched randomisation based on geography and radio listenership (figure 1). Specifically, we defined three radio listenership strata (61–70%, 71–80%, and >80%), and within each stratum we paired the areas geographically closest with each other, one of which was randomly assigned to receive the intervention. Randomisation was done by SS and SC, independently of DMI. The randomisation sequence was generated using computer-generated random numbers (Stata version 13). Because of time constraints, randomisation was done before the baseline survey. The nature of the intervention precluded formal masking of respondents and interviewers. The average number of villages included per cluster was 34 in the control group and 29 in the intervention group. In all clusters the government was the main health service provider and, with the exception of Kantchari (intervention cluster), a regional or district hospital was located in the town with the community radio station. The trial population also had access to primary health facilities in villages across each cluster.

### Interventions

DMI's radio campaign launched in March, 2012, and ended in January, 2015. A description of the theory of change and the Saturation+ methodology used to design and implement the campaign is provided elsewhere.^12^ Short spots, of 1 min duration, were broadcast approximately ten times per day, and 2 h, interactive long-format programmes were broadcast 5 days per week. All materials were produced in the predominant local languages spoken in each intervention cluster. The dramas were based on message briefs that DMI drew up for each target behaviour. The long-format programmes were followed by phone-ins to allow listeners to comment on the issues raised. Behaviours covered by spots changed weekly, while the long-format programme covered two behaviours a day and changed daily. Table 1 shows the campaign resources allocated to each of the target behaviours.

**Table 1:** Target behaviours and broadcasting intensity up to the month preceding the endline survey (October, 2014)

	**Number of weeks of spots**	**Number of long format modules**
**Maternal health**		
Four or more antenatal consultations	6	63
Saving during pregnancy*	6	63
Health facility delivery	10	61
**Newborn health**		
Breastfeeding initiation within 1 h after birth	8	45
First bath delayed for 24 h or more after birth in low birthweight†	1	11
**Child nutrition**		
Exclusive breastfeeding in 0–5 month-old children	12	102
Complementary feeding in 6–11 month-old children†	5	31
Growth monitoring in 0–23 month-old children†	4	28
**Health care-seeking for childhood illnesses**		
Health care-seeking for fever	23	100
Health care-seeking for pneumonia	13	84
Health care-seeking for diarrhoea	21	139
Oral rehydration salt or increase in fluids for diarrhoea‡	21	139
**Bednet**		
Bednet use in under-5 children and pregnant women†	6	144
**Sanitation**		
Safe disposal of child's stool†	3	94
Household latrine ownership†‡	2	94
Handwashing with soap	11	90

* Submessage for antenatal consultations.

† Spots no longer broadcast from midline.

‡ Submessage for care seeking for diarrhoea; three submessages for safe disposal of children's stool.

During the trial period, no other radio campaigns related to child survival and of comparable intensity were broadcast in any of the clusters included in the trial. Various health programmes operated in similar numbers of clusters per group (appendix p 1). From 2010 to 2013, community case management for malaria, pneumonia, and diarrhoea was supported by the Catalytic Initiative to Save a Million Lives in one of the intervention clusters and one of the control clusters, although the independent evaluation of this rapid scale-up programme concluded that it did not result in changes in coverage or mortality.^13^

### Cross-sectional household surveys

Cross-sectional household surveys were performed in all clusters at three time points: at baseline, from December, 2011, to February, 2012; at midline, in November, 2013, after 20 months of campaigning; and at endline, from November, 2014 to March, 2015, after 32 months of campaigning.

At baseline and endline surveys, a census of villages selected for the survey was performed with GPS coordinates recorded. For all households with at least one woman aged 15–49 years, the household head was interviewed to collect socioeconomic data and all women aged 15–49 years were interviewed on their pregnancy history. At baseline, due to time and cost constraints, the census took place in a simple random sample of villages covering half of the population in each cluster (about 20 000 inhabitants) and pregnancy history data collection was truncated to cover the period from January, 2005, to the date of the interview. At endline, the census took place in all villages included in the trial and a full pregnancy history was recorded.

At each survey, about 5000 mothers with at least one child younger than 5 years living with them were interviewed regarding their demographic characteristics, radio listenership, and family behaviours of relevance to child survival.^9^ To test recognition of the campaign at midline and endline, the two spots broadcast in the last 2 weeks of the previous month were played at the end of the interview and women were asked whether they had heard them on the radio. With respect to long format programmes, recognition was tested by referring to its title. At baseline and endline, mothers for the behavioural interviews were selected using systematic random sampling of all women interviewed about their pregnancy history. At midline, a two-stage sampling procedure was used.^9^

Before each survey, fieldworkers received 2 weeks' training. At baseline and endline, 84 fieldworkers were deployed across clusters in teams of six fieldworkers. At midline, 56 fieldworkers were deployed in teams of four fieldworkers. Each team was managed by a supervisor. Interviews were performed using Trimble Juno SB Personal Digital Assistants using Pendragon forms software. Data were backed up twice a week by a team of seven data managers and checked for consistency and completeness. Re-interviews were requested in cases of missing or inconsistent responses (for 7% of pregnancy history interviews and 3% of behavioural interviews at endline).

The trial was designed to detect a 20% reduction in the primary outcome (all-cause post-neonatal under-5 child mortality) with a power of 80%.^8^ We assumed a baseline mortality rate of 25 per 1000 per year, a coefficient of variation between clusters of 0·18, that mortality would decline in all clusters by 5% over the course of the study, and that the analysis would be based on cluster-level summaries with adjustment for pre-intervention mortality. Simulations indicated that, given a total of 14 clusters, a sample size of 7000 under-5 children per cluster would be required. The sample size of 5000 mothers was calculated assuming a design effect of 2 with a view to providing an absolute precision of within 10% or better for all behaviours.

### Routine health facility data

Routine health facility data from January, 2011, to February, 2016, were obtained from the Direction Générale des Etudes et des Statistiques Sanitaires of the Ministry of Health. For 78 primary health facilities located in trial clusters (41 in control clusters), monthly numbers were provided for: pregnant women attending for a first antenatal consultation, facility deliveries, and all-cause under-5 child consultations.

### Outcomes

The primary outcome was all-cause post-neonatal under-5 child mortality, the secondary outcome was all-cause under-5 child mortality, and intermediate outcomes included the coverage of the campaign (as measured by the proportion of mothers who reported listening to the campaign) and family behaviours targeted by the campaign as listed in table 1 (as measured by the proportion of mothers who reported a given behaviour during interviews and the number of attendances at primary health facilities).

### Statistical analysis

Primary analyses were performed on an intention-to-treat basis, and followed an analysis plan agreed in advance with the trial's Independent Scientific Advisory Committee. All analyses were performed on cluster-level summary measures^14^ and adjusted for pre-intervention levels to control for imbalances between groups and improve precision. The matching procedure was ignored, as recommended for trials with fewer than ten clusters per group.^15^ All clusters were given equal weight in all analyses.

All analyses were done with Stata (versions 13 and 14).

### Analysis of mortality

**Figure 2:**
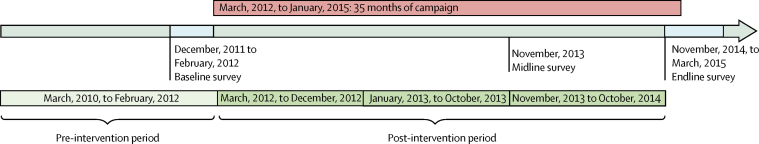
Pre-intervention and post-intervention periods for mortality analysis

The pre-intervention period was defined as the 2 years before the campaign, from March, 2010, to February, 2012. The post-intervention (March, 2012, to October, 2014) period was split into three periods: from March, 2012, to December, 2012 (the first 10 months of campaigning), January, 2013, to October, 2013 (next 10 months), and November, 2013, to October, 2014 (the 12 months preceding the start of the endline survey; figure 2). Full pregnancy history data collected at the endline survey were used to calculate both pre-intervention and post-intervention cluster-level mortality estimates. Cluster-level estimates of post-neonatal under-5 child mortality and under-5 child mortality were computed using the Demographic and Health Survey (DHS) synthetic cohort life-table approach. Missing months of birth (for 2% of livebirths across all pre-intervention and post-intervention periods) were randomly imputed according to the DHS method.^16^ This method relies on the construction of logical ranges for each date, which are refined in three steps, resulting in successively narrower ranges. In the final step, months of birth are randomly imputed within the final constrained logical range.

An analysis of covariance was performed on a log-risk scale to estimate the risk ratio for the effect of the intervention adjusted for pre-intervention mortality. The Wild bootstrap test, recommended when there are few clusters,^17^ was used to test for evidence of an intervention effect and for evidence of effect modification by post-intervention period.

### Analysis of change from baseline in self-reported behaviours

We did a cluster-level difference-in-difference analysis to assess the change from baseline to follow-up survey (either at midline or endline) in self-reported behaviours. For each of the 17 target behaviours, cluster-level differences in prevalence from baseline to follow-up survey were calculated and regressed on the intervention status of clusters and the cluster-level baseline prevalence to account for regression to the mean. Wild bootstrap tests were done to test the null hypothesis of no intervention effect. No formal adjustment was made to account for multiple testing. Analyses of maternal and newborn related behaviours at midline and endline were restricted to pregnancies ending after June, 2012 (with at least 4 months of exposure to the campaign).

### Adjustment for confounder score

At baseline, the mean post-neonatal under-5 mortality risk during the 2 years preceding the intervention was estimated at 112·3 per 1000 children in the intervention group versus 82·9 per 1000 children in the control group. Three covariates, expected to predict mortality, were particularly imbalanced between groups at baseline: the distance to the capital, Ouagadougou, as a proxy for general level of development (158 km in the control group *vs* 232 km in the intervention group); the median distance to the closest health facility (2·5 km *vs* 6·3 km, respectively); and the facility delivery prevalence (82% *vs* 56%, respectively). These covariates were combined using principal component analysis to produce a single cluster-level summary confounder score. After controlling for the confounder score, the pre-intervention mortality risk difference between groups estimated at baseline was reduced from 30·9 to 6·8 per 1000 children. To control for imbalance between groups, analyses of behaviour and mortality were adjusted for the confounder score.

### Effect modification

Three categories of radio ownership were defined: no radio in the compound (or household), radio in the compound but not in the household, and radio in the household. The Wild bootstrap test was used to test for evidence of effect modification by radio ownership.

With respect to care-seeking behaviours, three categories of distance to the closest health facility were also defined (<2 km, 2–5 km, and >5 km) to look for evidence of effect modification by distance on service-dependent behaviours, using the same analysis as described above.

### Analyses of routine health facility data

In a first analysis, the absolute number of attendances was calculated by yearly period (March, 2011, to February, 2016) and by cluster. For each post-intervention period, the ratio of the absolute number of attendances over the absolute number in the year before the intervention was then calculated in each cluster, a mean ratio to baseline was then computed by group, and a Wild bootstrap test, adjusted for confounder score, was used to compare the mean ratio to baseline between groups.

In addition, an interrupted time-series analysis was also done using mixed-effects Poisson regression of monthly counts of attendances per cluster, from January, 2011, to February, 2016. The model included fixed effects allowing for a long-term secular trend, for month of the year to account for seasonal variation, for intervention status of the cluster to account for systematic differences between groups at baseline, for confounder score, and for intervention effect by period, with cluster treated as a random effect.^18^ To obtain 95% CI and p values, we used bootstrap resampling (using the BC_a_ method and 1000 bootstrap replications).^19^

This trial is registered with ClinicalTrial.gov, number NCT01517230.

### Role of the funding source

The funders of the study had no role in study design, in the collection, analysis, and interpretation of data, in the writing of the report, and in the decision to submit the paper for publication. The corresponding author had full access to all the data in the study and had final responsibility for the decision to submit for publication.

## Results

**Figure 3:**
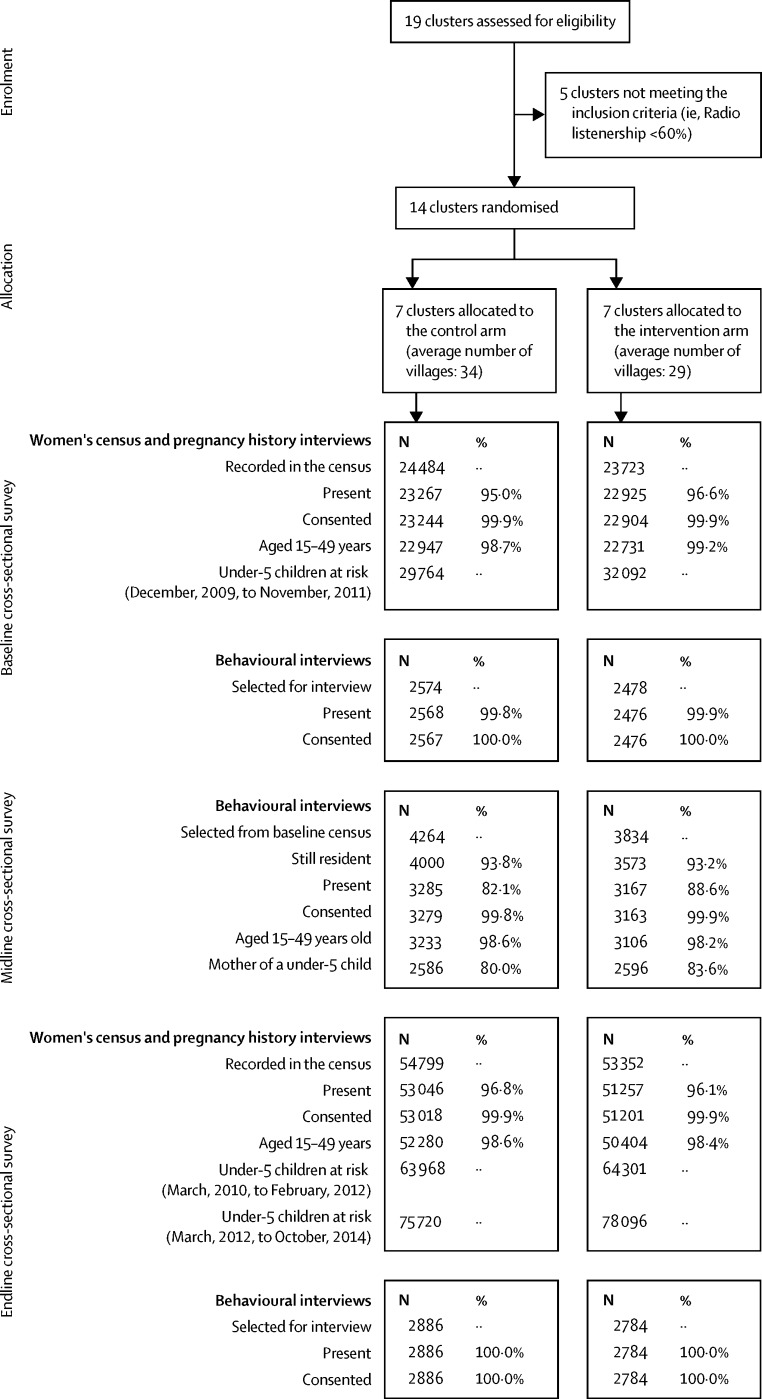
Trial profile

Pregnancy histories were completed for 102 684 women aged 15 to 49 years at endline (figure 3). At baseline and endline, respectively, the behavioural questionnaire was completed for 5043 and 5670 mothers of a child younger than 5 years and living with them across the 14 clusters.

Baseline sociodemographic characteristics have been reported in detail elsewhere.^7^ Briefly, while many characteristics were similar across groups at baseline, there were some important differences with respect to ethnicity, religion, and distance to the closest health facility (table 2). There was little change in sociodemographic characteristics between surveys.

**Table 2:** Mothers' sociodemographic characteristics

		Control group baseline*(N=2567)	Control group endline*(N=2886)	**Intervention group baseline**†**(N=2476)**	**Intervention group endline**†**(N=2784)**
Age (years)		28·9 (7·2)	28·3 (7·1)	28·4 (7·1)	27·6 (6·8)
3 years or more residence duration in the village		2339 (91%)	2656 (93%)	2319 (94%)	2624 (95%)
Ethnicity					
	Mossi	1077 (42%)	1343 (47%)	743 (30%)	840 (31%)
	Gourmantche	295 (12%)	343 (12%)	664 (27%)	731 (27%)
	Gourounssi	566 (22%)	575 (20%)	79 (3%)	94 (3%)
	Peulh	166 (7%)	171 (6%)	420 (17%)	522 (19%)
	Gouin, Karaboro, or Turka	6 (<1%)	0	342 (14%)	316 (12%)
	Marka, Dafing, or Dioula	214 (8%)	268 (9%)	87 (4%)	82 (3%)
	Bwaba or Bobo	191 (8%)	123 (4%)	82 (3%)	105 (4%)
	Other	42 (2%)	43 (2%)	52 (2%)	62 (2%)
Religion					
	Muslim	1209 (47%)	1430 (50%)	1482 (60%)	1743 (63%)
	Catholic or protestant	1154 (45%)	1305 (46%)	652 (26%)	669 (24%)
	Animist	199 (8%)	132 (5%)	333 (14%)	340 (12%)
School attendance		400 (16%)	583 (20%)	251 (10%)	396 (14%)
Household socioeconomic status					
	1 (poorest)	362 (14%)	378 (13%)	463 (19%)	487 (18%)
	2	428 (17%)	459 (16%)	502 (20%)	556 (20%)
	3	494 (19%)	550 (19%)	500 (20%)	571 (21%)
	4	555 (22%)	672 (24%)	495 (20%)	545 (20%)
	5 (least poor)	719 (28%)	796 (28%)	498 (20%)	609 (22%)
Radio ownership					
	No radio	524 (21%)	597 (21%)	325 (13%)	483 (18%)
	Radio in the compound	429 (17%)	514 (18%)	543 (22%)	678 (25%)
	Radio in the household	1606 (63%)	1747 (61%)	1589 (65%)	1607 (58%)
Married		2488 (97%)	2778 (98%)	2428 (98%)	2690 (98%)
Polygamous union		984 (40%)	932 (34%)	978 (40%)	1111 (41%)
Two or more under-5 children		1005 (39%)	1218 (43%)	1141 (46%)	1316 (48%)
Age of the youngest child in months		21·1 (14·8)	18·8 (13·7)	19·4 (13·9)	18·5 (13·4)
Distance to the nearest health facility					
	<2 km	1014 (40%)	1045 (36%)	454 (18%)	497 (18%)
	2–5 km	851 (33%)	991 (34%)	699 (28%)	654 (24%)
	>5 km	702 (27%)	850 (30%)	1323 (53%)	1633 (59%)

Data are mean (SD) or n (%).

* At baseline: from 0% to 3·1% missing values across variables; at endline: from 0% to 3·7%.

† At baseline: from 0% to 1·9% missing values across variables; at endline: from 0% to 3·4%.

**Figure 4:**
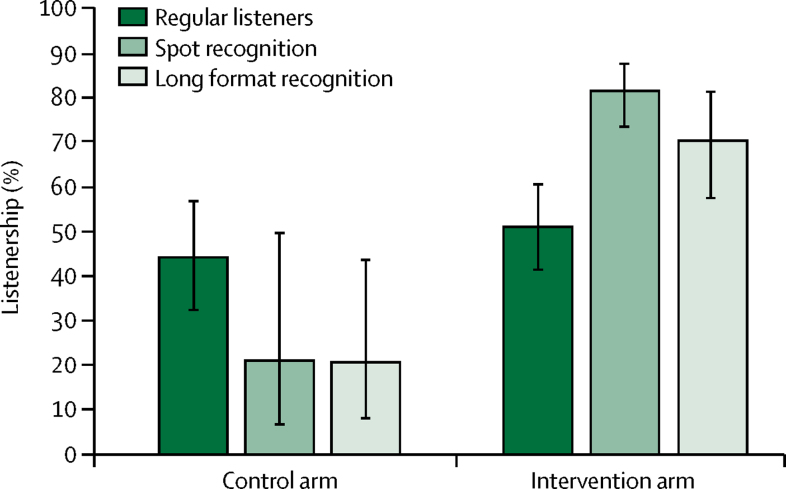
Radio listenership and campaign recognition at endline Error bars represent 95% CI.

Household radio ownership was similar in both groups at baseline, about 60%, and changed little at endline (table 2). Across surveys, women's radio listenership in the past week averaged 52% (at baseline: 1309 [53%] of 2472; at endline: 1419 [51%] of 2784) in the intervention clusters and 46% in the control clusters (at baseline: 1246 [49%] of 2567; at endline: 1274 [44%] of 2886; figure 4). In the intervention clusters, listenership, in the past week, to the radio station that was broadcasting the intervention varied from 74% (678/913) in March, 2011, prior to the implementation, to 43% (1208/2784) at endline, reflecting possible seasonal variation. Contamination was reported in one control cluster with, respectively at midline and endline, 33% (124/375) and 37% (148/397) of women in the Gayeri control cluster reporting having listened in the past week to the campaign's partner radio station in the neighbouring Bogande intervention cluster (figure 1). At endline, 2269 (82%) of 2784 interviewed women in the intervention group reported recognising spots played at the end of the interview and 1968 (71%) reported listening to a long format programme. In the control group, around 20% of women also reported recognising spots and long format programmes (606 [21%] and 594 [21%] of 2884, respectively). When asked on which radio station they listened to these broadcasts, 1782 (79%) of 2269 women mentioned DMI's radio partners in the intervention group compared with 208 (34%) of 606 women in the control group (13 [5%] of 268 excluding the Gayeri control cluster where “contamination” occurred).

Before the campaign (March, 2010, to February, 2012), the post-neonatal under-5 child mortality risk was 93·3 per 1000 livebirths (95% CI 72·5–114·2) in the control group versus 125·1 per 1000 livebirths (95% CI 104·8–145·5) in the intervention group (table 3). The between-cluster coefficient of variation was 0·34 across all clusters. We recorded similar substantial decreases in risk in both groups over time, to 58·5 per 1000 livebirths (95% CI 44·8–72·3) in the control group versus 85·1 per 1000 livebirths (67·7–102·6) in the intervention group during the last period (November, 2013, to October, 2014). After controlling for pre-intervention mortality and confounder score, there was no evidence of an intervention effect (risk ratio [RR] 1·00, 95% CI 0·82–1·22; p >0·999) across the intervention period. There was no suggestion that the effect of the intervention increased or decreased over time (p=0·353). Results were similar for under-5 child mortality (table 4).

**Table 3:** Effect of Development Media International's radio campaign on all-cause post-neonatal under-5 child mortality (intention-to-treat analysis)

	**Mortality rate per 1000 livebirths (95% CI)**		**Risk ratio (95% CI); p value**	
	Control group	Intervention group	Cluster-level analysis, adjusted for pre-intervention level*	Cluster-level analysis, adjusted for pre-intervention level and confounder score†
March, 2010, to February, 2012	93·3 (72·5–114·2)	125·1 (104·8–145·5)	..	..
March, 2012, to December, 2012	88·0 (66·3–109·8)	114·5 (95·5–133·5)	0·99 (0·83–1·18); 0·870	1·00 (0·81–1·24); 0·919
January, 2013, to October, 2013	71·4 (52·4–90·4)	105·2 (81·9–128·4)	1·00 (0·82–1·22); 0·991	0·95 (0·76–1·18); 0·640
November, 2013, to October, 2014	58·5 (44·8–72·3)	85·1 (67·7–102·6)	1·06 (0·84–1·32); 0·591	1·04 (0·80–1·36); 0·729
March, 2012, to October, 2014	71·8 (53·5–90·2)	100·5 (82·0–119·0)	1·01 (0·86–1·20); 0·842	1·00 (0·82–1·22); 0·999

* p value for effect modification by time=0·353.

† p value for effect modification by time=0·353.

**Table 4:** Effect of Development Media International's radio campaign on all-cause under-5 child mortality (intention-to-treat analysis)

	**Mortality rate per 1000 livebirths (95% CI)**		**Risk ratio (95% CI); p**	
	Control group	Intervention group	Cluster-level analysis, adjusted for pre-intervention level*	Cluster-level analysis, adjusted for pre-intervention level and confounder score†
March, 2010, to February, 2012	115·5 (93·6–137·4)	150·5 (126·8–174·1)	..	..
March, 2012, to December, 2012	105·0 (81·8–128·3)	137·0 (115·6–158·3)	1·02 (0·85–1·23); 0·763	1·03 (0·83–1·29); 0·560
January, 2013, to October, 2013	87·9 (66·9–108·9)	126·5 (102·8–150·2)	1·06 (0·92–1·21); 0·310	1·01 (0·87–1·18); 0·846
November, 2013, to October, 2014	76·5 (60·1–92·8)	105·1 (85·0–125·1)	1·04 (0·82–1·32); 0·696	1·02 (0·77–1·36); 0·843
March, 2012, to October, 2014	89·1 (68·8–109·3)	121·6 (101·4–141·9)	1·04 (0·88–1·22); 0·534	1·02 (0·84–1·24); 0·710

* p value for effect modification by time=0·698.

† p value for effect modification by time=0·698.

At baseline, most service-dependent behaviours tended to be reported more commonly in the control group than in the intervention group, while home-based behaviours were more similar between groups (appendix p 2). In both groups, the proportion of children who had fever, fast or difficult breathing, or diarrhoea in the 2 weeks preceding the interview and who were reported to have received appropriate treatment was quite low at a third or less. We noted a similar low prevalence for early breastfeeding initiation and sanitation-related behaviours (appendix p 2). Other home-based behaviours were more common, reported by about 40–60% of mothers. We previously reported some evidence, at midline, of an effect of the intervention on self-reported appropriate family responses to diarrhoea and fast or difficult breathing, and on saving during the pregnancy.^7^ At endline, the only self-reported behaviour for which there was some evidence of an intervention effect was saving during the pregnancy (baseline prevalence and confounder-score-adjusted difference-in-difference 14·2%, 95% CI 2·4–25·9; p=0·053; appendix p 2). For the other target behaviours, baseline prevalence and confounder-score-adjusted difference-in-differences ranged from −11·3% (95% CI −34·4 to 11·8) for recommended antimalarials for fever to 22·0% for breastfeeding initiation within 1 h after birth (95% CI −14·4 to 58·5; p>0·330).

**Figure 5:**
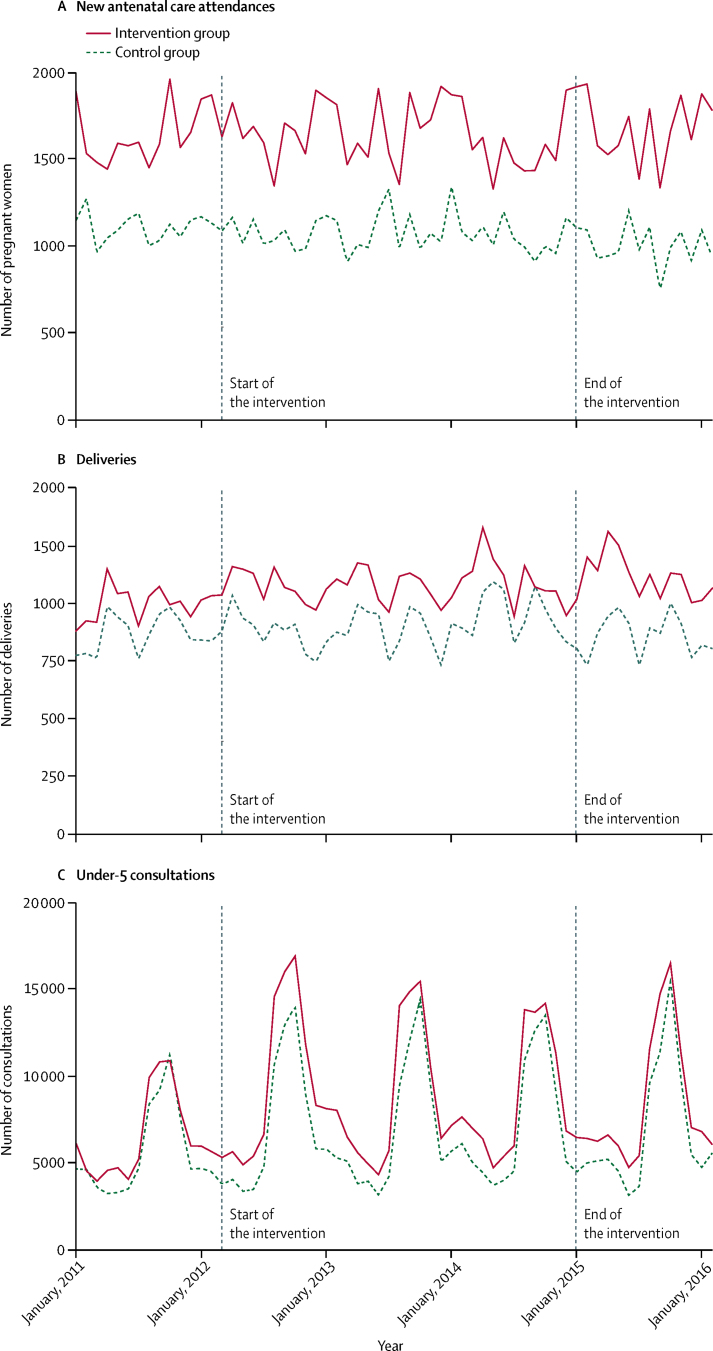
(A) New antenatal care attendances, (B) deliveries, and (C) under-5 consultations at primary facilities by month and by group (routine facility data)

Table 5 summarises the absolute numbers of attendances in primary facilities located in trial clusters for antenatal care consultations, deliveries, and under-5 consultations by group and time period. Figure 5 shows the same data by month. New antenatal care attendances remained relatively constant in both groups over the entire study period (table 5). Facility deliveries seemed to increase slightly in the intervention group while remaining relatively constant in the control group. Under-5 consultations increased in the first year of the intervention by 40% in the intervention group compared with 21% in the control group. In the second and third years of the intervention, the number of consultations remained steady in the control group and fell slightly in the intervention group. Despite a much larger increase in attendances in the intervention group, a simple analysis based on cluster-level summaries did not provide any statistical evidence for an intervention effect.

**Table 5:** Absolute numbers of attendances at primary facilities by time period and by group (routine facility data)

	**New ANC attendances, control group**	**New ANC attendances, intervention group**	p value*	**Deliveries, control group**	**Deliveries, intervention group**	p value*	**Under-5 consultations, control group**	**Under-5 consultations, intervention group**	p value*
March, 2011, to February, 2012	13 129 (1·00)	19 658 (1·00)	..	10 598 (1·00)	12 155 (1·00)	..	68 681 (1·00)	79 852 (1·00)	..
March, 2012, to February, 2013	12 997 (0·99)	20 202 (1·03)	0·439	10 533 (0·99)	12 902 (1·06)	0·787	83 022 (1·21)	111 758 (1·40)	0·853
March, 2013, to February, 2014	13 129 (1·00)	20 340 (1·03)	0·469	10 688 (1·01)	12 896 (1·06)	0·866	82 559 (1·20)	103 191 (1·29)	0·323
March, 2014, to February, 2015	12 627 (0·96)	19 332 (0·98)	0·564	11 117 (1·05)	13 228 (1·09)	0·901	82 528 (1·20)	102 257 (1·28)	0·291
March, 2015, to February, 2016	11 902 (0·91)	19 768 (1·01)	0·240	10 505 (0·99)	13 353 (1·10)	0·558	83 873 (1·22)	103 136 (1·29)	0·403

Data are n (ratio to baseline). ANC=antenatal care.

* p value for the comparison of the mean ratio to baseline between groups.

Table 6 shows the estimates of the intervention effect by period computed from the interrupted time-series analysis. In the first year of the campaign, there were small increases in new antenatal care attendances (6%; p=0·004) and deliveries (7%; p=0·004) in the intervention group compared with the control group. We noted a substantial increase in under-5 consultations (35%; p<0·0001) in the intervention group. In the second and third years, the estimated effect on new attendances to antenatal care and deliveries remained relatively constant, although without statistical evidence for the former in year 3. The effect on under-5 consultations seemed to decrease over time, but evidence of an intervention effect remained (year 2: 20%; p=0·003, year 3: 16%; p=0·049).

**Table 6:** Intervention effect by time period on attendances at primary facilities (routine facility data)

	**New ANC attendances**	**Deliveries**	**Under-5 consultations**
First year of the intervention (March, 2012, to February, 2013)	1·06 (1·02–1·10); 0·004	1·07 (1·02–1·11); 0·004	1·35 (1·20–1·51); <0·0001
Second year of the intervention (March, 2013, to February, 2014)	1·09 (1·01–1·18); 0·026	1·06 (1·02–1·11); 0·003	1·20 (1·06–1·37); 0·003
Third year of the intervention (March, 2014, to February, 2015)	1·08 (0·98–1·18); 0·129	1·09 (1·04–1·14); <0·0001	1·16 (1·00–1·35); 0·049
Post-intervention period (March, 2015, to February, 2016)	1·11 (0·99–1·25); 0·081	1·09 (1·01–1·17); 0·023	1·12 (0·92–1·37); 0·272

Data are risk ratio (95% CI); p value. ANC=antenatal care.

There was no evidence that the effect of the intervention on post-neonatal under-5 child mortality varied with radio ownership after controlling for pre-intervention mortality and confounder score (p=0·164; appendix p 3). With respect to self-reported behaviours, we only did tests for effect modification on care-seeking behaviours for childhood illness (to avoid performing a multiplicity of tests) to investigate whether patterns were consistent with the routine health facility data. There was no evidence for effect modification by radio ownership after controlling for baseline prevalence and confounder score (p=0·204; appendix p 4).

We noted strong evidence that the effect of the intervention on self-reported care-seeking behaviours for childhood illness varied with distance to the closest health facility, with baseline prevalence and confounder-score-adjusted difference-in-differences of around 23% and 14% for families within 2 km and 2–5 km from a facility, respectively, compared with an estimated difference-in-difference of −13·7% among those living further away (p=0·004; appendix p 4).

## Discussion

We found no evidence of an effect of a mass media campaign on child mortality. This finding comes against a background of rapidly decreasing mortality in both groups, which will have reduced our power to detect an effect on mortality (set at 80% to detect a 20% reduction in mortality). The decrease in mortality we recorded is broadly consistent with estimates for Burkina Faso as a whole from the UN Inter-Agency Group for Child Mortality Estimation (IGME). Recent improvements in child survival could reflect changes in national health policies, in particular two rounds of free national distribution of insecticide-treated bednets (2010 and 2013), and the addition of the pneumococcal and rotavirus vaccines to the expanded programme for immunisation in 2013. However, routine health facility data did provide evidence of increased utilisation of health services in intervention clusters relative to control clusters, especially with respect to care seeking for childhood illness. Self-reported behaviours might have been over-reported due to socially desirable bias, especially in the intervention group as a consequence of DMI's campaign itself. Nevertheless, we observed some evidence of improved care seeking and treatment in the midline survey.^9^ Although no overall difference was apparent at the endline survey, the survey data are consistent with increased care seeking among families living within up to 5 km of a facility, with no effect at greater distances.

With only a limited number of clusters available, a major limitation of our trial is that, despite randomisation, important differences between the intervention and control groups at baseline were not unlikely.^14^ The use of pre-intervention mortality estimated at baseline survey was precluded by the intervention timeframe, and we therefore used a pair-matched randomisation procedure based on geography and estimated radio listenership. Nevertheless, intervention communities had a different ethnic and religious mix, tended to live further away from health facilities, and experienced higher mortality than the control communities. We generated a confounder score to account for imbalance between groups, but cannot exclude the possibility of residual confounding. Furthermore, contamination of one of the control areas occurred due to an increase in the strength of the transmission signal of the neighbouring radio partner, above that permitted by the national authorities. However, excluding women living in villages where contamination occurred had little effect on the results (data not shown).

The DMI campaign seems to have reached a high proportion of the primary target population as a high proportion of mothers interviewed in the intervention group reported recognising DMI's spots and listening to the long format programmes. One in five women in the control clusters also reported recognising the spots or long format programme. Excluding the control cluster in which contamination occurred, only a few women mentioned one of DMI's radio partners when asked on which radio station they listened to these broadcasts, which could suggest courtesy bias or confusion with other radio programmes.

In interpreting these results it should be considered that our survey data are likely to have much lower power than the facility data to detect a change in care seeking. While the survey data include 1000 or fewer sick children per group, the facility data record tens of thousands of consultations. However, both sources of data are prone to errors. Retrospective reporting of illness episodes and care seeking in surveys is known to have important limitations. We used a recall period of 2 weeks, as used in DHS, but it has been shown that recall of disease episodes tends to decline after a few days,^20^, ^21^, ^22^, ^23^, ^24^ as well as reporting of clinic visits.^20^ Thus, our population-based surveys almost certainly missed some episodes of recent illness. However, the routine facility records might also be subject to recording errors and come without precise and up-to-date denominator data. The population of Burkina Faso is estimated to be increasing by about 3% per year^11^ and it is therefore likely that the under-5 child population served by the facilities for which we have data was increasing over time. Interpretation of the observed differences between intervention and control groups as being attributable to the intervention requires the assumption that any increases in the underlying populations served by the facilities were of similar magnitude in both groups (or smaller in the intervention group). However, we have no reason to believe that population growth differed between groups.

The facility data suggest a large increase in under-5 consultations in the intervention group in the first year of the intervention. The estimated impacts in subsequent years are smaller. While this apparent decline could be a chance finding, it might reflect attenuation in the effect of the intervention. In Burkina Faso, in-depth interviews with health workers and patients have revealed low satisfaction with the quality of care in public facilities.^25^, ^26^ The low use of and dissatisfaction with community-based insurance in northwest Burkina Faso has been attributed, in part, to the suboptimal quality of care provided, including poor health worker attitudes and behaviours.^27^ In the same area, Mugisha and colleagues found that, while many factors influence initiation of the demand for services, only perceived quality of care predicted “retention” in modern health-care services.^28^ They concluded that increasing patient initiation and patient retention require different interventions and that the latter should focus on improving the perceived quality of care. A possible, admittedly speculative, explanation for our findings is that women were initially encouraged by the campaign to take their children to a facility, but that poor perceived quality of care may have discouraged some from returning for subsequent illnesses.

Our findings showed no effect of the campaign on self-reported habitual behaviours, such as child feeding practices, handwashing, and child stool disposal practices. The campaign's broadcasts were heavily weighted to care seeking rather than home-based behaviours, and as we have discussed previously, it might be harder to achieve sustained changes in habitual behaviours that need to be performed daily with little obvious immediate benefit, than for behaviours that are only performed occasionally and for which some immediate benefit may be perceived.^9^ The confidence intervals for the effect of the intervention on habitual behaviours are wide and do not preclude modest but important changes in these behaviours.

While we detected evidence that the intervention was associated with an increase in care-seeking in facilities we did not detect any evidence of a reduction in mortality. There are several possible explanations for this apparent inconsistency. First, our mortality data do not exclude the possibility of an impact on mortality with the lower bounds of the 95% confidence interval for the mortality risk ratio compatible with an important reduction in mortality. The impact of the campaign on child mortality has been modelled using the Lives Saved Tool and showed an estimated 8% reduction in the first year, and 5% reduction in the second and third years (unpublished data). In addition, mortality at baseline differed between the two groups despite randomisation. Although we adjusted for pre-intervention mortality risk and a confounder score, which performed reasonably well in explaining the baseline mortality imbalance, we cannot exclude the possibility of residual confounding which might have masked an intervention effect. Second, while the numbers of consultations with diagnoses of malaria, pneumonia and diarrhoea, three of the leading causes of child death in Burkina Faso, all increased (unpublished data), we have no data on the severity of the episodes for which children were taken to facilities. If most of the increase in consultations was due to children with mild self-limiting illness, then limited impact on mortality might be expected. In some parts of Burkina Faso a preference for traditional care has been reported for some severe manifestations of illness, such as cerebral malaria.^29^, ^30^ Third, if the quality of care received at the facility was low, this could limit any mortality reduction through increased care seeking. An evaluation of the quality of care at health facilities for children under-5, conducted in 2011 in two regions in the north of Burkina Faso, found that on average only six of ten tasks that should be performed as part of IMCI were performed.^31^ Only 28% of children were checked for three danger signs, and 40% of children judged to require referral by an Integrated Management of Childhood Illness expert were referred by the health worker. In addition, the 2010 DHS indicated that among children in rural areas who were taken to a public primary health facility, 54% of those with fever received an antimalarial, 35% of those with diarrhoea received oral rehydration solution, and 77% of those with cough and fast or difficult breathing received an antibiotic. Fourth, mortality data were collected by interviewing women about their pregnancy histories. Such data are subject to measurement errors. We did a number of checks on the data, similar to those routinely performed by DHS. Apart from heaping of deaths at age 12 months, which occurred to a similar degree in both groups and should not have affected the under-5 (post-neonatal) mortality estimates, these analyses did not identify any major concerns. The cluster-level estimates of mortality risk at baseline correlated well with subnational estimates from the 2010 DHS and the time trend in mortality is broadly consistent with that estimated by the UN Inter-agency Group for Child Mortality Estimation.

In summary, there is evidence that DMI's campaign led to increased use of health facilities, especially by sick children. However, we noted no effect of the campaign on child mortality. The small number of clusters available for randomisation together with the substantial between-cluster heterogeneity at baseline, and rapidly decreasing mortality, limited the power of the study to detect modest changes in behaviour or mortality. Caution should be exercised in interpreting these results since, despite randomisation, there were important differences between intervention and control clusters at baseline. Nevertheless, this study provides some of the best evidence available that a mass media campaign alone can increase health facility utilisation for maternal and child health in a low-income, rural setting.
