# Supplementary material for: Effect of a mass radio campaign on family behaviours and child survival in Burkina Faso: a repeated cross-sectional, cluster-randomised trial
Source: Lancet Glob Health. 2018 Feb 9;6(3):e330–41. doi: 10.1016/S2214-109X(18)30004-4 (PMC5817351; doi:10.1016/S2214-109X(18)30004-4)
Supplement: Supplementary appendix [file mmc1.pdf]

THE LANCET
Global Health

Supplementary appendix

This appendix formed part of the original submission and has been peer reviewed. We post it as supplied by the authors.

Supplement to: Sarrassat S, Meda N, Badolo H, et al. Effect of a mass radio campaign on family behaviours and child survival in Burkina Faso: a repeated cross-sectional, cluster-randomised trial. *Lancet Glob Health* 2018; **6:** e330–41.

Table 1: Other major programs implemented in intervention and control clusters

Cluster	International NGOs / programs	# local OBCE
Banfora	CREPA; Progetto Mondo MLAL	6
Bogande	ACF; GRET (Nutrifaso program); Helvetas (Sanifaso program); UNICEF	6
Djibo	Croix Rouge; CRUS; DRC; Hunger Project	3
Kantchari	ACF	1
Ouahigouya	-	5
Sapouy	Hunger Project; MSI; PAM; UNICEF	0
Solenzo	SOS Sahel	2
All intervention clusters	ACF; CREPA; Croix Rouge; CRUS; DRC; Hunger Project; MSI; GRET (Nutrifaso program); PAM; Progetto Mondo MLAL; Helvetas (Sanifaso program); SOS Sahel; UNICEF	23
Boromo	Water Aid (Sanifaso program)	4
Gayeri	HKI; GRET (Nutrifaso program); PAM	3
Kongoussi	Plan International (PAASAD program); SOS Sahel	5
Koudougou	GIZ; PAM; Water Aid (Sanifaso program); USAID	5
Nouna	MSI; PAM; SOS Sahel	4
Po	-	3
Pouytenga	Medicus Mundi; Plan International (PAASAD program)	3
All control clusters	GIZ; HKI; Medicus Mundi; MSI; GRET (Nutrifaso program); PAM; Plan International (PAASAD program); SOS Sahel; USAID; Water Aid (Sanifaso program)	27

Table 2: Changes from baseline in self-reported behaviours (ITT analysis)

	Survey	Control arm total	Control arm %	Intervention arm total	Intervention arm %	"Crude" DiD	Cluster-level DiD analysis: Adjusted for baseline prevalence DiD	95%CI		P-value	Adjusted for baseline prevalence and confounder score DiD	95%CI		P-value
Maternal health														
4 or more ANC visits	BS	2,562	**50.8**	2,470	**37.0**									
	ES ITT	2,378	**56.4**	2,361	**41.7**	-0.9	**-7.9**	-23.0	7.3	0.440	**-4.8**	-23.8	14.1	0.729
Saving during the pregnancy	BS	2,562	**62.8**	2,474	**56.4**									
	ES ITT	2,378	**63.2**	2,361	**63.9**	7.1	**8.9**	-0.9	18.7	0.061	**14.2**	2.4	25.9	0.053
Health facility delivery	BS	2,562	**81.8**	2,470	**56.0**									
	ES ITT	2,378	**89.7**	2,361	**69.8**	5.9	**4.9**	-14.1	23.9	0.515	**3.3**	-16.9	23.5	0.638
Newborn health														
Breastfeeding initiation within 1 hour after birth	BS	2,556	**32.6**	2,463	**26.8**									
	ES ITT	2,342	**32.6**	2,316	**35.4**	8.6	**7.7**	-15.0	30.3	0.491	**22.0**	-14.4	58.5	0.360
First bath delayed for 24 hours or more after birth	BS	2,556	**55.9**	2,463	**49.3**									
	ES ITT	2,358	**50.8**	2,331	**39.2**	-5.0	**-3.2**	-11.2	4.9	0.398	**-1.2**	-12.8	10.5	0.722
Health care seeking in a health facility or with a CHW (two weeks prior to interview)														
Fever, fast/difficult breathing or diarrhoea	BS	1,072	**57.4**	975	**43.2**									
	ES ITT	822	**62.2**	830	**51.6**	3.6	**1.9**	-18.5	22.4	0.812	**0.6**	-22.9	24.2	0.931
Fever	BS	735	**63.7**	637	**50.2**									
	ES ITT	582	**73.5**	572	**58.2**	-1.8	**-0.3**	-18.3	17.7	0.973	**-3.2**	-24.7	18.2	0.716
Fast/difficult breathing	BS	302	**56.6**	381	**44.4**									
	ES ITT	281	**54.1**	244	**41.8**	-0.1	**-5.0**	-27.3	17.3	0.656	**-8.3**	-38.1	21.4	0.532
Diarrhoea	BS	559	**57.8**	514	**44.9**									
	ES ITT	357	**64.4**	370	**54.1**	2.6	**2.6**	-16.0	21.2	0.750	**-0.9**	-22.8	21.0	0.923
Treatment (two weeks prior to interview)														
ACT or quinine IM/IV for fever	BS	735	**17.3**	639	**16.9**									
	ES ITT	613	**39.3**	587	**27.6**	-11.3	**-11.3**	-27.6	4.9	0.131	**-11.3**	-34.4	11.8	0.472
Antibiotic for fast/difficult breathing	BS	302	**28.2**	382	**27.0**									
	ES ITT	284	**35.6**	244	**25.8**	-8.6	**-8.5**	-33.7	16.6	0.488	**-8.3**	-44.2	27.5	0.884
ORS or more liquids for diarrhoea	BS	560	**41.1**	516	**30.6**									
	ES ITT	371	**49.6**	376	**39.6**	0.5	**-8.2**	-18.0	1.6	0.062	**-10.4**	-22.8	1.9	0.471
Home-made solutions for diarrhoea	BS	560	**6.8**	516	**6.8**									
	ES ITT	371	**9.7**	376	**4.0**	-5.7	**-4.8**	-18.2	8.6	0.608	**-15.8**	-30.8	-0.7	0.297

Nutrition														
Exclusive breastfeeding (day prior to interview, 0-5 months old)	BS	429	**44.6**	450	**42.4**									
	ES ITT	525	**54.9**	490	**58.8**	6.1	**7.8**	-11.9	27.4	0.423	**6.1**	-21.6	33.7	0.586
Complementary feeding (day prior to interview, 6-11 months old)	BS	418	**55.5**	411	**49.9**									
	ES ITT	546	**63.6**	536	**60.1**	2.1	**-0.2**	-17.2	16.7	0.982	**-11.2**	-30.7	8.3	0.330
Growth monitoring (past 6 months, 0-23 months old)	BS	1,525	**59.7**	1,615	**51.1**									
	ES ITT	1,940	**68.3**	1,904	**51.1**	-8.6	**-9.4**	-22.5	3.6	0.145	**-7.7**	-24.9	9.6	0.462
Bed net use														
Children under an ITN the night prior to interview	BS	2,567	**60.3**	2,475	**58.5**									
	ES ITT	2,886	**68.2**	2,784	**68.1**	1.7	**-0.1**	-10.6	10.3	0.972	**-1.2**	-15.7	13.2	0.854
Women under a net during their last pregnancy	BS	2,560	**65.6**	2,468	**62.5**									
	ES ITT	2,886	**67.4**	2,784	**68.9**	4.6	**1.7**	-11.3	14.8	0.798	**3.6**	-14.6	21.8	0.671
Sanitation														
Household latrine ownership	BS	2,559	**19.5**	2,458	**25.0**									
	ES ITT	2,858	**40.6**	2,768	**39.9**	-6.2	**-6.8**	-24.2	10.6	0.396	**4.2**	-18.7	27.1	0.675
Safe disposal of last children's stools †	BS	2,566	**14.1**	2,475	**15.3**									
	ES ITT	2,886	**21.7**	2,784	**21.6**	-1.3	**-1.2**	-6.5	4.0	0.640	**1.1**	-6.3	8.5	0.849
HWWS the last time women cleaned her child who defecated	BS	2,535	**37.5**	2,401	**36.4**									
	ES ITT	2,733	**42.6**	2,688	**44.1**	2.6	**1.7**	-8.6	12.1	0.713	**-2.2**	-16.0	11.6	0.595

BS: Baseline survey; ES: Endline survey; ITT: Intention-To-Treat analysis

† defined when the child used a latrine or when the stool was thrown into them or buried

Table 3: Effect modification by radio ownership on all-cause post-neonatal under-five child mortality (ITT analysis)

Period	Control arm			Intervention arm			Cluster-level analysis: Adjusted for pre-intervention level			P-value †	Cluster-level analysis: Adjusted for pre-intervention level and confounder score			P-value †
	Risk	95% CI		Risk	95% CI		RR	95% CI			RR	95% CI		
No radio														
Mar 2010 - Feb 2012	**92.1**	64.9	119.3	**127.6**	105.2	150.0	-	-	-		-	-	-	
Mar 2012 - Oct 2014	**80.9**	58.7	103.2	**99.9**	81.1	118.8	**0.63**	0.34	1.17	0.164	**0.63**	0.30	1.31	0.164
Radio in the compound, not in the household														
Mar 2010 - Feb 2012	**90.3**	75.5	105.0	**123.7**	91.8	155.6	-	-	-		-	-	-	
Mar 2012 - Oct 2014	**79.3**	62.4	96.2	**108.8**	81.4	136.1	**0.98**	0.76	1.27		**0.92**	0.69	1.24	
Radio in the household														
Mar 2010 - Feb 2012	**94.7**	70.4	119.0	**124.7**	103.6	145.9	-	-	-		-	-	-	
Mar 2012 - Oct 2014	**66.3**	49.1	83.5	**97.5**	81.8	113.2	**1.07**	0.88	1.30		**1.02**	0.82	1.28	

† P-value for effect modification by radio ownership

Table 4: Effect modification by radio ownership on self-reported care seeking (ITT analysis)

	Survey	Control arm		Intervention arm		"Crude" DiD	Cluster-level DiD analysis: Adjusted for baseline prevalence			P-value †	Cluster-level DiD analysis: Adjusted for baseline prevalence and confounder score			P-value †
		Total	%	Total	%		DiD	95% CI			DiD	95% CI		
	No radio													
Care seeking in a health facility or with CHW for fever, fast/difficult breathing or diarrhoea	BS	219	**55.3**	119	**43.7**	-	-	-	-		-	-	-	
	ES ITT	180	**68.9**	142	**45.8**	-11.5	**-5.9**	-32.8	21.0	0.204	**-5.6**	-31.5	20.3	0.204
	Radio in the compound, not in the household													
	BS	164	**53.1**	236	**44.5**	-	-	-	-		-	-	-	
	ES ITT	157	**64.3**	209	**45.9**	-9.8	**-7.8**	-35.2	19.6		**-7.5**	-31.9	16.9	
	Radio in the household													
	BS	687	**59.1**	618	**42.7**	-	-	-	-		-	-	-	
	ES ITT	476	**59.2**	475	**55.8**	13.0	**7.8**	-10.7	26.3		**8.1**	-12.5	28.7	

† P-value for effect modification by radio ownership

Table 5: Effect modification by distance to the closest facility on self-reported care seeking (ITT analysis)

	Survey	Control arm		Intervention arm		"Crude" DiD	Cluster-level DiD analysis: Adjusted for baseline prevalence			P-value †	Cluster-level DiD analysis: Adjusted for baseline prevalence and confounder score			P-value †
		Total	%	Total	%		DiD	95% CI			DiD	95% CI		
	< 2 km													
Care seeking in a CSPS for fever, fast/difficult breathing or diarrhoea	BS	386	**64.5**	180	**43.3**	-	-	-	-		-	-	-	
	ES ITT	300	**61.7**	133	**58.7**	18.2	**17.2**	-4.4	38.8	0.003	**22.7**	-1.8	47.1	0.004
	2 - 5 km													
	BS	353	**49.3**	277	**40.4**	-	-	-	-		-	-	-	
	ES ITT	298	**54.0**	199	**51.8**	6.7	**8.7**	-9.2	26.5		**14.1**	-5.5	33.7	
	> 5 km													
	BS	333	**28.2**	518	**37.6**	-	-	-	-		-	-	-	
	ES ITT	224	**49.6**	498	**41.2**	-17.8	**-20.1**	-44.2	4.1		**-13.7**	-39.1	11.8	

† P-value for effect modification by distance to the closest facility
